# Single‐cell RNA‐seq reveals altered NK cell subsets and reduced levels of cytotoxic molecules in patients with ankylosing spondylitis

**DOI:** 10.1111/jcmm.17159

**Published:** 2022-01-06

**Authors:** Conglin Ren, Mingshuang Li, Yang Zheng, Bingbing Cai, Weibin Du, Helou Zhang, Fengqing Wu, Mengsha Tong, Fu Lin, Jinfu Wang, Renfu Quan

**Affiliations:** ^1^ The Third Clinical Medical College of Zhejiang Chinese Medical University Hangzhou Zhejiang China; ^2^ The First Affiliated Hospital of Zhejiang Chinese Medical University Hangzhou Zhejiang China; ^3^ Research Institute of Orthopedics The Affiliated Jiangnan Hospital of Zhejiang Chinese Medical University Hangzhou Zhejiang China; ^4^ Department of Orthopedics Hangzhou Xiaoshan Hospital of Traditional Chinese Medicine Hangzhou Zhejiang China; ^5^ Institute of Cell and Development Biology, College of Life Sciences Zhejiang University Hangzhou Zhejiang China

**Keywords:** ankylosing spondylitis, granzyme B, natural killer cells, peripheral blood mononuclear cells, single‐cell RNA sequencing

## Abstract

Ankylosing spondylitis (AS) is an autoimmune disease with unknown aetiology. To unravel the mechanisms mediating AS pathogenesis, we profiled peripheral blood mononuclear cells (PBMCs) from AS patients and healthy subjects using 10X single‐cell RNA sequencing. The frequencies of immune cell subsets were evaluated by flow cytometry. NK cells were purified from PBMCs using isolation kit and were examined for gene expression by RT‐qPCR. Plasma levels of cytolytic molecules were examined by enzyme‐linked immunosorbent assay. Compared to healthy controls, AS patients showed a significant decrease in total NK cells as well as CD56^dim^ NK subset, whereas CD56^bright^ NK cells were increased. Additionally, impaired expression of cytotoxic genes in NK cells of AS patients was observed by bioinformatics algorithm and verified by RT‐qPCR and flow cytometry. Consistent with changes in transcriptomics, we found decreased plasma levels of granzymes, but not granulysin, in AS patients. Furthermore, Pearson correlation analysis revealed a negative correlation between plasma GZMB levels and disease activity (*r* = −0.5275, *p* = 0.0358). No correlation was observed between plasma cytolytic molecules and biochemical indexes (ESR and CRP). Our findings uncover altered NK cell subsets and cytotoxic profiles in peripheral circulation of AS patients at single‐cell resolution.

## 1 INTRODUCTION

Ankylosing spondylitis (AS) is a branch of spondyloarthritis (SpA), characterized by long‐term rheumatic inflammation of unknown origin. The current diagnostic system tends to classify SpA into two categories: peripheral SpA (mainly affecting the extremities, associated with psoriasis, inflammatory bowel disease or preceding infection) and axial SpA (mainly affecting the spine, such as AS).^1^ Chronic inflammatory back pain is a characteristic complaint of most patients with AS, presenting as a dull or vague ache that often worsens in the morning or evening.^2^ Over time, osteophyte formation and ligament ossification induced by prolonged inflammatory irritation contribute to irreversible structural damage.^3^ In some severe cases, complete fusion of the spine causes kyphosis, limited thoracic mobility and complications such as cardiopulmonary and digestive dysfunction. For now, there is no cure for AS, but reasonable physical exercise and specific medication, such as JAK inhibitor, can effectively alleviate pain and prevent the condition from worsening.^4^, ^5^

Although there is no satisfactory explanation for the disease mechanism, many hypotheses have been proposed from clinical practice or animal experiments. HLA‐B27 is present in up to 70–80% of patients and its association with AS is still considered to be important.^6^ Unlike other HLA‐B alleles, due to the specificity of the molecular structure, heavy chain of HLA‐B27 readily forms dimers and oligomers that not only bind to immune‐related receptors (e.g. KIR3DL2) on the cell surface but also trigger unfolded protein responses, resulting in inflammatory responses.^7^ Imbalance of immune cell populations is also thought to be a hallmark of AS.^8^ Previous studies have shown that the proportion of CD4 lineage T cell subsets, such as Th17 and Th22, that secrete inflammatory cytokines is elevated in the peripheral circulation of AS patients.^9^, ^10^ In another line of research, some scholars linked AS to the changes in cytotoxic cell profiles. A large‐scale genotyping of immune‐related loci involving 10,619 AS cases and 15,145 controls identified four CD8^+^ lymphocyte‐associated SNPs, including EOMES, IL7R, RUNX3 and ZMIZ1.^11^ By analyzing epigenetic, RNA sequencing and protein expression data, Li et al.^12^ showed that AS‐associated loci were enriched in immune cell types (e.g., monocytes and T cells) and that proteins encoded by genes downregulated in AS patients were enriched in CD8^+^ T cells and natural killer (NK) cells. Gracey et al.^13^ found a changed cytotoxic cell profile in AS patients whose joint fluid had a significantly increased number of CD8^+^ T cells with activated phenotype. In addition to AS, studies of other diseases in SpA family (such as psoriatic arthritis and SpA with Crohn’s disease) consistently support a pathogenic role for CD8^+^ T cells, suggesting that cytotoxicity‐related factors may play a neglected role in the development of SpA.^14^, ^15^ Despite the updated knowledge of AS in recent years, research on the role of NK cells in the pathogenesis and pathophysiology of AS has been less satisfactory.

Understanding gene expression differences between phenotypes is crucial for transcriptomics studies. As a novel method for reliable assessment of transcript abundance, single‐cell RNA sequencing (scRNA‐seq) focuses on gene expression in individual cells, thus providing higher resolution and better understanding of the status of different cell populations. Here, we isolated peripheral blood mononuclear cells (PBMCs) from AS patients and healthy controls and examined a subset of participants (*n* = 3 per group) using 10X scRNA‐seq technology to explore the heterogeneity in immune cell populations and cytotoxic profiles between the two groups. Pilot data generated from scRNA‐seq were followed up with flow cytometry, RT‐qPCR and enzyme‐linked immunosorbent assay (ELISA) in our larger cohort.

## 2 MATERIAL AND METHODS

### 2.1 Study subjects

In this study, two cohorts totaling 29 AS patients and 29 healthy controls were enrolled. Detailed characteristics of the participants were summarized in Table S1. All patients with AS fulfilled the modified New York criteria, and active disease was defined by Bath Ankylosing Spondylitis Disease Activity Index (BASDAI) ≥4.^16^ This study was approved by the Ethics Committees of Hangzhou Xiaoshan Hospital of Traditional Chinese Medicine. All participants were informed of the details of this experiment and signed consent forms.

### 2.2 Isolation of PBMCs from fresh peripheral blood

We isolated PBMCs using Lymphoprep density gradient medium (#07801, Stem Cell Technologies) according to the user guide. Then, enriched PBMCs were washed twice with PBS and stained with 0.4% Trypan blue solution (#PB180423, Procell Life Science & Technology) to assess cell viability.

### 2.3 cDNA library construction and 10X single‐cell sequencing

Briefly, single cells, reverse transcription reagents, Gel Beads containing barcoded oligonucleotides and oil were combined on a microfluidic chip to form Gel Bead‐in‐Emulsions. Subsequently, libraries (comprising standard Illumina paired‐end constructs, Read 1, and Read 2) were constructed using Chromium Single Cell 3′ gene expression kit (version 3.1). With the help of Gene Denovo Technology, we performed 10X single‐cell sequencing on Illumina NovaSeq 6000 at a sequencing depth of ~50,000 reads per cell.

### 2.4 Data processing pipelines

After sequencing, the original BCL files were converted to FASTQ files using Cell Ranger (version 3.1.0). Upon completion of gene expression quantification, we transferred the output data to Seurat R package (version 3.2.3) for subsequent analysis.^17^

To further exclude unwanted cells, we set strict quality control criteria: the number of genes identified in a single cell was between 500 and 4000; the number of UMIs in a single cell <20,000 and the percentage of mitochondrial gene expression in a single cell <10%. Principal component analysis was performed to reduce the number of gene dimensions, and UMAP (uniform manifold approximation and projection) algorithm was run to visualize cells in a two‐dimensional space. Thereafter, marker genes of each cluster were identified using “FindAllMarkers” function embedded in Seurat R package. To become a cluster‐specific differentially expressed gene (DEG), there were three conditions to be met. First, |log2 fold‐change| ≥0.25. Second, the gene was expressed in more than 25% of cells in the target cluster. Third, adjusted *p*‐value ≤0.05. Cell‐type annotation was automatically inferred by “SingleR” package^18^ and then manually checked according to known cell‐linage‐specific genes.

### 2.5 Function enrichment analysis of marker genes

Gene Ontology (GO) and KEGG are internationally standardized databases that identify enriched biological functions and pathways by comparison with genome‐wide background. We used clusterProfiler R package^19^ in RStudio (version 1.2.1335) and ClueGO plugin^20^ in Cytoscape software (version 3.8.2) for GO and KEGG analysis, respectively. Results with adjusted *p*‐value <0.05 were retained according to the hypergeometric test algorithm.

### 2.6 Flow cytometry

Peripheral blood mononuclear cells were isolated from fresh blood and resuspended at 10 × 10^6^/ml in flow cytometry staining buffer (#420201, BioLegend). Then, cells were incubated with fluorescent conjugated antibodies, including APC‐Cy7‐CD3 (#557757, BD Pharmingen) and BV421‐CD56 (#562751, BD Pharmingen), on ice for 20 min in the dark. To detect the expression of intracellular cytotoxic molecules, cells were fixed and permeabilized using Fixation and Permeabilization Buffer Set (#88‐8824, eBioscience), and then stained with GZMB antibody (#515406, BioLegend) or IgG1 κ (#400136, BioLegend) as an isotype control. After washing twice by centrifugation at 350 ***g*** for 5 min, cells were resuspended in 0.5 ml of staining buffer and harvested using CytoFlex S (Beckman Coulter). Data analysis was performed using FlowJo software (version 10).

### 2.7 Isolation of NK cells and RNA extraction

Natural killer cells were harvested by using human NK cell isolation kit (#17955, Stem Cell Technologies). To assess the efficiency of NK cell sorting, enriched cells were analyzed by flow cytometry. Total RNA was extracted from NK cells using RNA‐quick purification kit (#RN001, Yi Shan Biotechnology), followed by RNA quality assay (Nanodrop, Thermo Scientific) and first‐strand cDNA synthesis (#K1622, Thermo Scientific). Amplification of cDNA was performed on ABI‐7500 system (Applied Biosystems). Primer sequences for RT‐qPCR were shown in Table S2. The results of gene expression were calculated using the $2^{‐\Delta \Delta C_{T}}$ method.

### 2.8 Detection of cytolytic molecules in plasma

Plasma levels of cytotoxic granules, including GZMA, GZMB and granulysin, were quantified using ELISA kits (#EK1162, #EK1114 and # EK1280, BOSTER Biological Technology). Each plasma sample was diluted with equal volume of dilution buffer prior to testing, and duplicate analyses were performed to ensure the reliability of the assay.

### 2.9 Statistical analysis

Statistical analysis was performed using GraphPad Prism (version 7.0). Comparisons between AS patients and healthy controls were made using unpaired *t*‐test, and results were shown as mean ± SEM. Receiver operating characteristic (ROC) curve was adopted to estimate the predictive ability of a specific gene. Area under the ROC curve (AUC) >0.8 implies that the gene is able to distinguish between patients and healthy controls and may be a valuable diagnostic biomarker for AS. Correlations between plasma levels of cytotoxic particles and BASDAI were calculated using Pearson correlation analysis. All tests with *p*‐value <0.05 were considered significant.

## 3 RESULTS

### 3.1 scRNA‐seq identified major immune cell populations in peripheral blood

**FIGURE 1:**
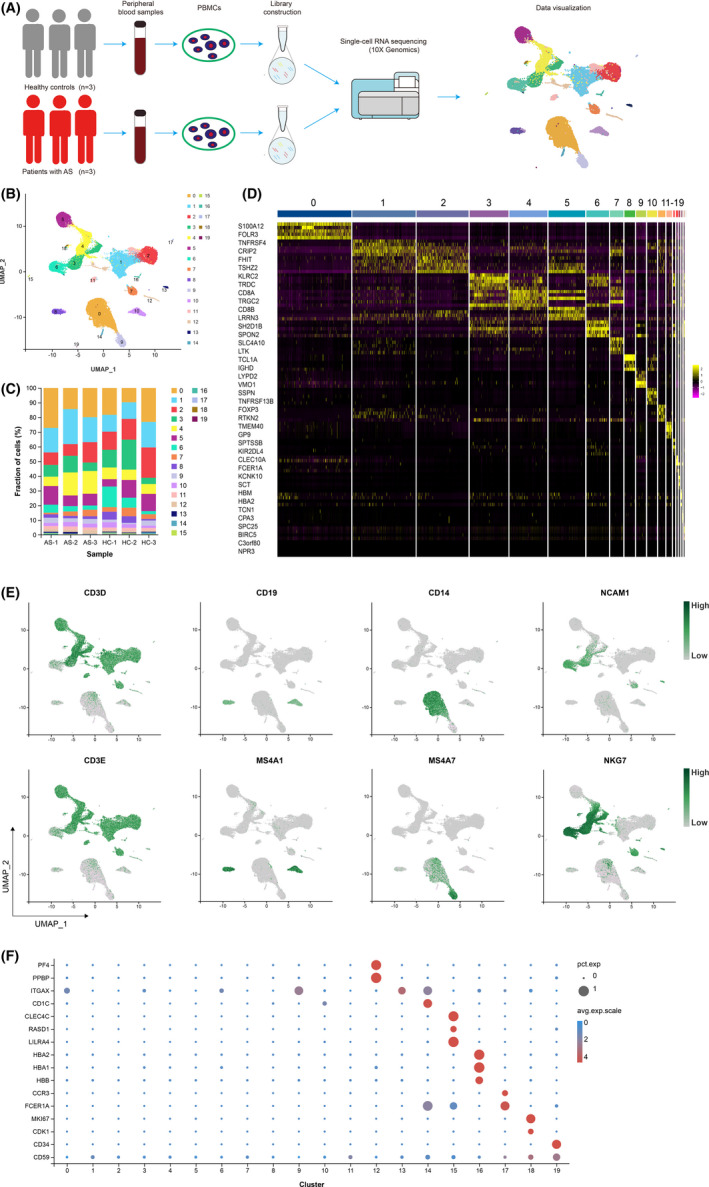
Single‐cell transcriptome profiling of PBMCs from AS patients (*n* = 3) and controls (*n* = 3). (A) Schematic diagram of the experimental workflow. (B) Two‐dimensional UMAP visualization of PBMCs resulted in 20 clusters. (C) The fractions of cell clusters in each sample. (D) Top 2 differentially expressed genes that were upregulated in each cluster were visualized in Heatmap. (E) Expression of marker genes for T cells, B cells, monocytes and NK cells (left to right). (F) Bubble plot shows the expression levels of key linage defining genes among all clusters. The size of bubble indicates the percentage of cells expressing a specific gene, and the colour of bubble indicates the average level of gene expression

We harvested PBMCs from three AS patients and three healthy subjects by gradient density centrifugation and profiled them based on 10X Genomics platform (Figure 1A). A total of 53,823 cells were recovered according to the quality control criteria mentioned in “Methods” section, of which 24,810 cells were from the AS group and 29,013 cells from the control (Table S3). The number of genes and UMIs detected per cell and the percentage of mitochondrial genes were all within the normal range (Figure S1). Unbiased clustering of PBMCs yielded 20 clusters, covering 10 different cell types that were identified based on classical surface markers of immune cells (Figure 1B, C, Table 1).

**TABLE 1:** Cell type and number of each cluster

Cluster	Cell type	Classical markers	Number of cells
0	Classical monocytes	CD14	9798
1	T cells	CD3D, CD3E	8797
2	T cells	CD3D, CD3E	7216
3	T cells	CD3D, CD3E	5500
4	T cells	CD3D, CD3E	5343
5	T cells	CD3D, CD3E	5176
6	Natural killer cells	NCAM1, NKG7	3208
7	T cells	CD3D, CD3E	1885
8	B cells	CD19, MS4A1	1436
9	Non‐classical monocytes	FCGR3A, MS4A7	1329
10	B cells	CD19, MS4A1	1317
11	T cells	CD3D, CD3E	1022
12	Megakaryocytes	PF4, PPBP	806
13	Natural killer cells	NCAM1, NKG7	327
14	Conventional dendritic cells	ITGAX	207
15	Plasmacytoid dendritic cells	CLEC4C, RASD1, LILRA4	156
16	Erythrocytes	HBA1, HBA2, HBB	103
17	Granulocytes	CCR3, FCER1A	90
18	MKI67^+^ proliferating cells	MKI67, CDK1	88
19	Haemopoietic stem cells	CD34, CD59	19

Specifically, T cells comprised seven clusters highly expressing CD3D and CD3E; B cells were characterized by CD19 and MS4A1 expression; classic and non‐classical monocytes were marked by the presence of CD14 and MS4A7, respectively; NK cells showed expression of NCAM1 (CD56) and lack of CD3 molecules (Figure 1D, E). Specific genes in cluster 12 include platelet‐associated factor PF4 and PPBP, both proteins belonging to the CXC family and released from the alpha granules of activated platelets, indicating that this cluster consists of megakaryocytes (Figure 1F). Commonly, dendritic cells are classified as “conventional dendritic cells (cDCs)” vs. “plasmacytoid dendritic cells (pDCs)”.^21^ Here, we used CD11C (ITGAX) to distinguish cDCs (cluster 14) and selected three markers (CLEC4C, RASD1 and LILRA4) to define pDCs (cluster 15) (Figure 1F). In addition, four small clusters were annotated as erythrocytes (cluster 16), granulocytes (cluster 17), MKI67+ proliferating cells (cluster 18) and haematopoietic stem cells (cluster 19) according to their respective markers (Figure 1F, Table 1). Apart from the widely known identity markers mentioned above, we obtained substantial novel cluster‐specific genes that will be informative for future single‐cell studies (Table S4).

### 3.2 NK cells were depleted in AS patients

To understand which cell population distinguish AS patients from healthy controls, we first attempted to compare the percentage of each cell type between the two groups. Due to the limited number of samples examined by scRNA‐seq, we failed to identify the cell types that were uniquely enriched in AS (Table S5). Previous evidence, however, suggests that patients with AS do have multiple immune perturbations or dysregulation, such as marked expansion of certain T cell subset^22^ and monocytes^23^ in the peripheral circulation.

**FIGURE 2:**
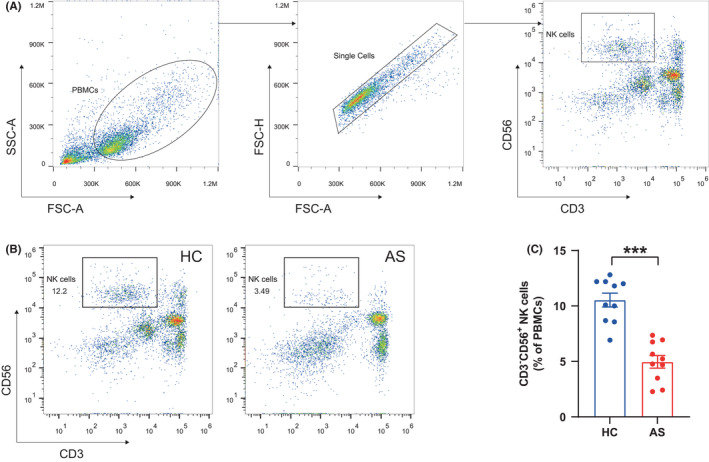
Reduction of total NK cells in peripheral blood of AS patients. (A) Gating strategy of CD3^−^CD56^+^ NK cells. (B) Representative flow cytometry plots showing CD3^−^CD56^+^ NK cells. (C) Proportions of CD3^−^CD56^+^ NK cells in PBMCs of healthy controls (HCs, *n* = 10) and AS patients (*n* = 10). Horizontal lines and error bars show the mean ± SEM. ***p < 0.001

In the current study, we mainly focused on NK cells and probed their alterations in AS condition, considering that the role of NK cells in the pathogenesis of AS is poorly understood but well studied in some other autoimmune diseases, such as systemic lupus erythematosus (SLE).^24^ For this purpose, we carried out flow cytometry and observed a significant reduction of CD3^−^CD56^+^ NK cells in PBMCs, with the mean percentage of NK cells in patients (4.96%) being much lower than in controls (10.53%) (*p* < 0.001) (Figure 2).

### 3.3 CD56^dim^ NK cell subset was reduced in AS patients

**FIGURE 3:**
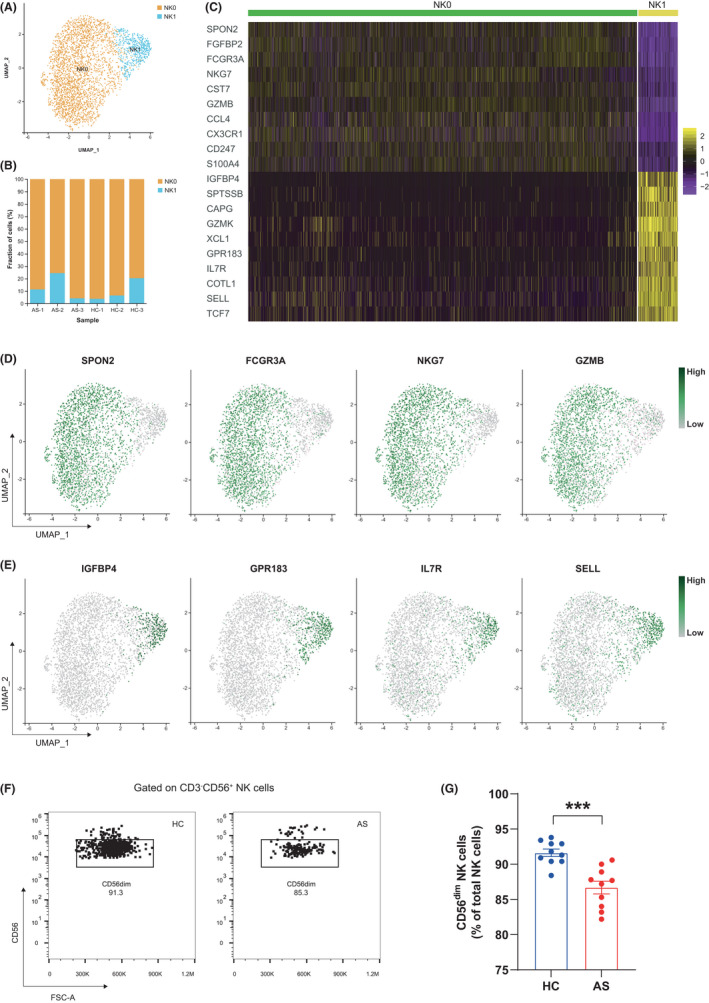
CD56^dim^ NK cell subset was diminished in AS patients. (A) Two‐dimensional UMAP visualization of NK cells resulted in two subsets. (B) The fractions of NK cell subsets in each sample. (C) Top 10 differentially expressed genes that were upregulated in each NK cell subset were visualized in Heatmap. (D) UMAP plots of the specific marker genes in NK0 subset (CD56^dim^). (E) UMAP plots of the specific marker genes in NK1 subset (CD56^bright^). (F) Representative flow cytometry plots showing CD56^dim^ NK cells. (G) Proportions of CD56^dim^ NK cells in total NK cells of HCs (*n* = 10) and AS patients (*n* = 10). Horizontal lines and error bars show the mean ± SEM. ***p < 0.001

Natural killer cells, also known as large granular lymphocytes, are cytotoxic lymphocytes that have the ability to recognize and kill harmful cells without the involvement of MHC molecules and antibodies and are therefore critical to the innate immune system. In the initial clustering atlas, clusters 6 and 13 were authenticated as NK cells (3535 in total), of which 1182 cells were from AS patients and 2353 cells were from controls (Figure 1B and Table S5). To analyse the NK cell population at a finer scale, we extracted all cells from clusters 6 and 13 and performed a secondary clustering analysis using the Seurat R package. As a result, two subsets of NK cells (named NK0 and NK1) were generated, exhibiting heterogeneous transcriptome properties with distinctive marker genes (Figure 3A, B and Table S6). In terms of the NK0 subset, the preferential expression of cytotoxic genes (such as GZMB and NKG7) as well as FCGR3A (an important receptor for initiating antibody‐dependent cellular cytotoxicity) implied a strong killing capacity, much like that previously reported for CD56^dim^ NK cells^25^ (Figure 3C, D). NK1 subset was not dominant in cell numbers; however, this subset was enriched in genes such as GPR183, IL7R, SELL and TCF7, which are important for lymphocyte activation, migration and functional regulation, indicating its identity as CD56^bright^ NK cells^26^ (Figure 3C, E).

Reviewing previous studies, we discovered that patients with certain immune diseases, such as SLE and multiple sclerosis, have dysfunctional NK cells.^27^ To see whether this condition exists in AS, we then compared the differences in NK cell composition between patients with AS and healthy controls. From Table S7 and Figure 3B, it seems that the NK compartment within AS patients was skewed towards CD56^bright^ (NK1) phenotype (except for the AS‐3 case). To follow‐up on this hypothesis, we quantified the circulating NK subsets in our larger cohort. Based on the flow cytometry results, we noted that the CD56^dim^ (NK0) subset was diminished in AS patients, with an average of 86.69% of total NK cells compared to 91.62% in healthy controls (*p* < 0.001) (Figure 3F, G).

### 3.4 Impaired expression of cytotoxic genes in NK cells of AS patients compared to healthy controls

**FIGURE 4:**
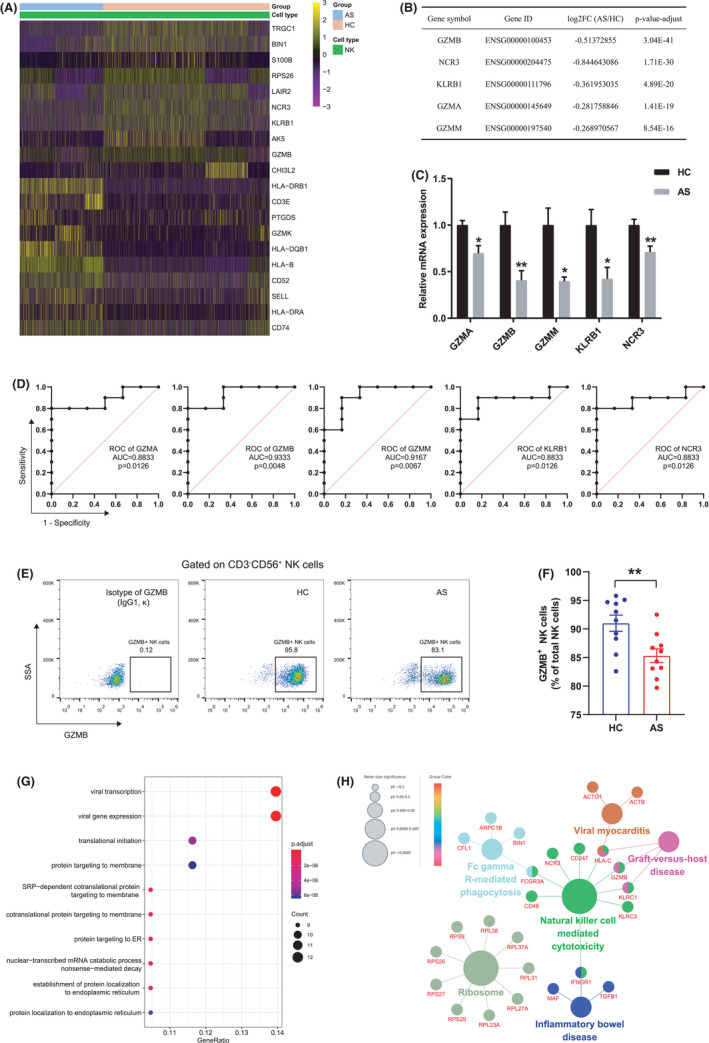
Impaired expression of cytotoxic genes in NK cells of AS patients. (A) Heatmap illustration of the representative up‐ and downregulated genes in NK cells from AS patients. (B) Top 5 downregulated cytotoxicity‐related molecules or receptors in AS patients vs. HCs. (C) RT‐qPCR analysis of gene expression fold changes in AS patients vs. HCs. (D) ROC curves were plotted to assess the ability of these five genes to differentiate between AS patients and HCs. (E) Representative flow cytometry plots showing GZMB^+^ NK cells. (F) Proportions of GZMB^+^ NK cells in total NK cells of HCs (*n* = 10) and AS patients (*n* = 10). (G) Top 10 biological processes for downregulated genes were shown in bubble plot according to gene ratio. (H) Use ClueGO plugin to analyse enriched KEGG pathways for downregulated genes. A gene involved in multiple pathways was presented with multiple colours. Horizontal lines and error bars show the mean ± SEM. *p < 0.05; **p < 0.01

To understand whether the reduction in NK cells was accompanied by altered transcripts, we analysed gene expression levels of NK cells between the two groups. Compared to NK cells from healthy controls, 114 genes were upregulated and 59 genes were downregulated in NK cells of AS patients (Table S8). Notably, genes associated with MHC molecules, which are known to be responsible for antigen presentation in immune responses, showed higher levels in AS patients (Figure 4A). In contrast, genes encoding cytotoxicity‐related molecules or receptors were at low levels, such as granzymes (GZMA, GZMB and GZMM) and killer cell lectin‐like receptors (KLRB1, KLRC1 and KLRC3); some transcription factors related to immunity and inflammatory regulation (e.g. CEBPB, MAF and JUNB) were also downregulated (Table S8). Among these DEGs, we mainly focused on changes in cytotoxic profiles and summarized top five cytotoxic genes in Figure 4B (ranked by adjusted *p*‐value). Moreover, we isolated NK cells from PBMCs of patients and healthy controls and compared the gene expression levels by RT‐qPCR. The tested up‐ and downregulated DEGs showed changes in the same direction as those observed by scRNA‐seq, with the most pronounced suppression of GZMB expression (Figure 4C and Figure S2A). ROC curves were plotted to assess the ability of these genes to differentiate between AS patients and healthy controls (Figure 4D). Consistent with the RT‐qPCR results, GZMB displayed optimal predictive power with AUC = 0.9333 (*p* = 0.0048). To verify the reduction of GZMB expression at the protein level, we performed flow cytometry analysis on AS patients and healthy controls (*n* = 10 in each group) by intracellular staining with GZMB antibody. Significantly, the percentage of GZMB^+^ NK cells in the total NK cell population was significantly lower in AS patients than in controls (*p* < 0.01) (Figure 4E, F).

We further studied the up‐ and downregulated DEGs by dropping them into GO and KEGG databases, respectively. As shown in Figure S2B, antigen processing and presentation, interferon‐γ response and T cell activation were significantly upregulated in patients with AS. By contrast, downregulated biological processes such as protein targeting to ER, protein targeting to membrane and translational initiation were identified (Figure 4G). Additionally, we evaluated affected KEGG pathways in AS patients. Consistent with GO analysis, the upregulated DEGs in NK cells from AS patients were enriched in antigen processing and presentation, T cell receptor signalling pathway and helper T cells differentiation (Figure S2C). For the downregulated DEGs, we noted that these genes were impactful in a number of pathways with immune or inflammatory context, and in particular, NK cell‐mediated cytotoxicity was hampered, which may result from impaired expression of cytotoxicity‐related genes encoding granzymes and NK cell receptors (Figure 4H).

### 3.5 Plasma levels of granzymes were declined in patients with AS

**FIGURE 5:**
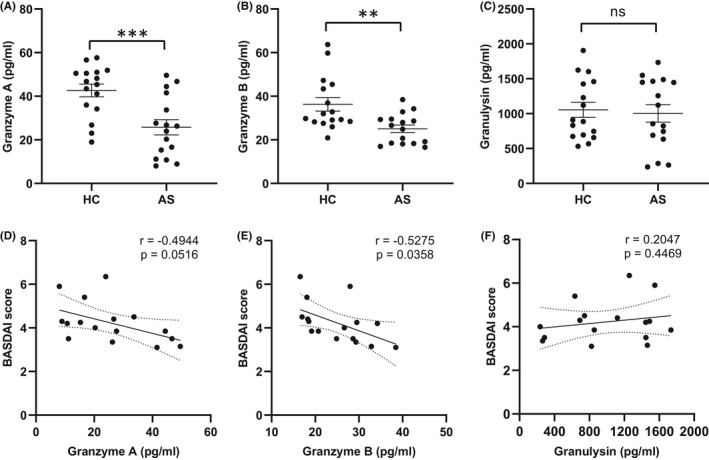
Expression of cytolytic molecules in plasma of HCs (*n* = 16) and AS patients (*n* = 16). The plasma levels of granzyme A (A), granzyme B (B) and granulysin (C) were determined by enzyme‐linked immunosorbent assay. Pearson correlation analysis was performed between granzyme A (D), granzyme B (E), granulysin (F) and disease activity (BASDAI). Horizontal lines and error bars show the mean ± SEM. **p < 0.01; ***p < 0.001; ns = not significant

To learn more about secretory cytolytic products in peripheral blood, we examined the plasma levels of GZMA, GZMB and granulysin in AS patients and healthy controls using ELISA kits. Consistent with changes in NK cell transcriptomics, AS patients had significantly lower protein levels of GZMA (25.73 ± 3.5 vs. 42.62 ± 2.94 pg/ml, *p* < 0.001) and GZMB (25.05 ± 1.73 vs. 36.28 ± 3.08 pg/ml, *p* < 0.01), but not granulysin (1003 ± 125.1 vs. 1055 ± 108.4 pg/ml, *p* = 0.7559), compared to healthy controls (Figure 5A–C). For AS patients, we further analysed the association between plasma cytolytic molecule levels and disease activity by means of Pearson’s algorithm. Plasma levels of GZMA and GZMB in AS patients were negatively correlated with BASDAI; however, only GZMB showed statistical significance (*r* = −0.5275, *p* = 0.0358) (Figure 5D, E). We did not observe either an association between plasma granulysin levels and BASDAI (Figure 5F) or correlations between the three cytolytic products and biochemical indexes (ESR and CRP) (data not shown).

## 4 DISCUSSION

In this study, we isolated PBMCs from peripheral blood and profiled them on the 10X Genomics platform. The primary clustering produced 20 clusters covering 10 cell types. Subsequently, we extracted NK cells from transcriptome data and performed secondary clustering to investigate the characteristics of NK subsets. Deep sequencing of RNA at single‐cell resolution uncovered remarkable heterogeneity in NK cell subsets and cytotoxic profiles between AS patients and healthy controls.

Natural killer cells can be involved in the entire process of initiation, progression and remission of inflammatory response, hence its role in autoimmune diseases is of great interest. Szántó et al.^28^ demonstrated an increase in NK cells in the peripheral circulation of AS patients; however, it was not replicated in our and other studies.^24^, ^29^ Our flow cytometry results supported a reduced frequency of total NK cells in patients with AS, accounting for 4.96% of PBMCs compared to 10.53% in healthy controls. This alteration was mainly attributed to the NK0 subset (CD56^dim^), as the percentage of NK1 subset (CD56^bright^) was actually elevated. Of note, inflammation in AS tends to be widespread and multiple, so the frequencies of NK cell subsets may vary among different specimens. Ciccia et al.^30^ analyzed NK cell subsets on ileal samples from 15 AS patients and 15 controls by flow cytometry and revealed an enrichment of NKp44^+^ but not NKp46^+^ NK cells in patients’ gut, which resulted in an overproduction of IL‐22. In other autoimmune diseases, Liu et al.^31^ revealed that CD56^dim^ NK subset in SLE patients showed a trend towards decreased proportion of the total NK cells and correlated with disease activity. Complementarily, the fraction of CD56^bright^ NK subset in peripheral blood of patients with active SLE was increased, accompanied by an accumulation of serum type I interferon levels.^32^ To delve into the properties of NK cells, Cosan et al.^33^ classified them into different types depending on cytokine secretion and found an increase in IFN‐γ^+^ NK cells in patients with Behcet's disease compared to controls, offset by a decrease in IL‐5^+^CD16^+^, IL‐17^+^CD16^+^ and IL‐10‐secreting regulatory NK subsets.

Previous microarray studies^34^, ^35^ demonstrated transcriptional heterogeneity between AS patients and healthy controls and captured a considerable number of DEGs, suggesting a multidimensional pathogenesis of this disease. However, these findings were based on whole blood profiling and failed to describe heterogeneity among distinct immune cell subset at a more precise level. Our scRNA‐seq provided novel insights by uncovering NK cell depletion in patients with AS, and then we asked whether this phenomenon was accompanied by altered transcripts. Compared with NK cells from healthy controls, we noted that the expression of cytotoxicity‐related molecules or receptors was hampered, including granzymes (GZMA, GZMB and GZMM) and NK cell receptors (such as NCR3 and KLRB1). Granzymes are a family of serine proteases containing five members in human, which was first proposed by Masson et al. in 1986.^36^ As the most widely studied member, GZMB is understood as an important mediator of tissue healing, chronic inflammation and immune response.^37^ An earlier study indicated that cytotoxic activity of NK cells and secretion of GZMB were reduced in systemic sclerosis patients, while proinflammatory cytokine secretion from NK cells was enhanced.^38^ Likewise, the insufficient number of NK precursors and downregulation of genes encoding perforin and granzymes resulted in lower cytotoxicity and lymphokine‐activated killer activity of NK cells from SLE patients.^24^ Additionally, the cytotoxicity of NK cells is regulated by a combination of signals from inhibitory and activating receptors. Jiao et al.^39^ investigated the polymorphisms in genes encoding NK cell receptors and found that KIR2DL1 and KIR2DL5 were more common in AS patients than in controls, which may impede the ability of NK cells to recognize and lyse target cells in immune response, thus contributing to the development of AS. KLRB1, perhaps better known by its alias CD161, is a member of the killer lectin‐like receptor family and widely expressed on the surface of NK and certain T cells. Data from a flow cytometry study highlighted that SLE patients had decreased levels of CD161 expression in both NK and T cells,^40^ and similar results were replicated by later investigators.^41^

The reduced expression of cytotoxicity‐related genes and skewing of the NK cell compartment towards CD56^bright^ phenotype (NK1) prompted us to question whether AS patients also exhibit altered circulating cytotoxicity. In our study, we found decreased plasma levels of granzymes (GZMA and GZMB) in AS patients using ELISA assay (no gross differences in granulysin). In agreement with our findings, Gracey et al.^13^ compared the expression of granzymes and perforin‐1 in serum, synovial fluid and mononuclear cells from healthy controls and patients with AS, osteoarthritis and rheumatoid arthritis and concluded that AS patients showed reduced expression of cytotoxic genes. Intriguingly, in their research, the lower levels of serum cytotoxic molecules were limited to perforin‐1 but not granzymes. When considering the inconsistency of altered cytotoxic profiles, there are two potential reasons: differences in ethnicity and geographic region and distinct assay methods (cytokine bead array vs. ELISA).

Our study has some limitations. We mainly focused on NK cells and cytotoxic profiles without detailing the transcriptome features of other members (e.g. T cells and monocytes) as well as changes in inflammatory cytokines. We are also aware of the limitations in sample source. Due to the scarcity of human skeletal and ligamentous specimens, we relied on peripheral blood throughout the study.

In conclusion, we demonstrate the differences in NK cell subsets and cytotoxic profiles between AS patients and healthy controls by 10X scRNA‐seq technology and experimental validation. Depletion of NK cells and impaired expression of cytotoxic molecules distinguish AS patients from healthy controls and are associated with disease activity (BASDAI), which may offer new insights for disease diagnosis and therapeutic intervention.

## CONFLICT OF INTEREST

The authors confirm that there are no conflicts of interest.

## AUTHOR CONTRIBUTIONS

**Conglin Ren:** Conceptualization (equal); methodology (equal); writing – original draft (equal). **Mingshuang Li:** Methodology (equal); writing – original draft (equal). **Yang Zheng:** Data curation (equal); software (equal). **Bingbing Cai:** Methodology (equal). **Weibin Du:** Funding acquisition (equal). **Helou Zhang:** Methodology (equal). **Fengqing Wu:** Validation (equal). **Mengsha Tong:** Validation (equal). **Fu Lin:** Validation (equal). **Jinfu Wang:** Conceptualization (equal); writing – review and editing (equal). **Renfu Quan:** Conceptualization (equal); supervision (equal); writing – review and editing (equal).

## Supporting information

Fig S1‐S2

Table S1‐S8

## Data Availability

The raw data of single‐cell RNA sequencing in this study have been deposited with the Sequence Read Archive (SRA) under accession number PRJNA749866.
