# Supplementary material for: Single‐cell RNA‐seq reveals altered NK cell subsets and reduced levels of cytotoxic molecules in patients with ankylosing spondylitis
Source: J Cell Mol Med. 2022 Jan 6;26(4):1071–82. doi: 10.1111/jcmm.17159 (PMC8831943; doi:10.1111/jcmm.17159)
Supplement: Supplementary file 1 — Fig S1‐S2 [file JCMM-26-1071-s001.pdf]

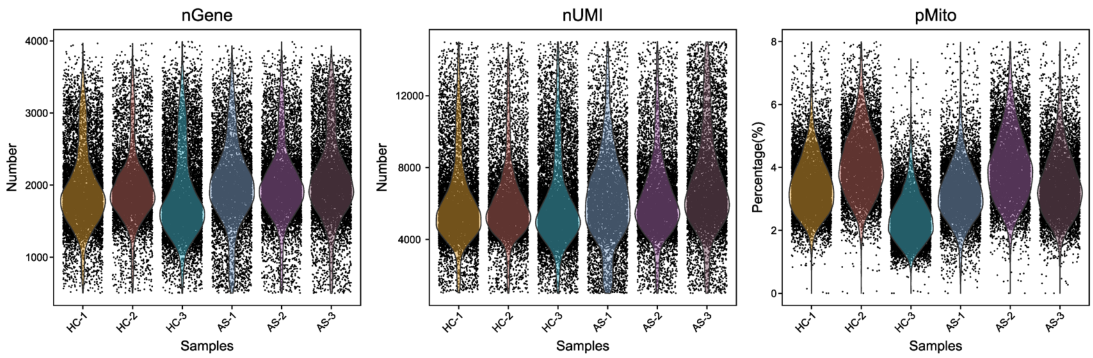

Figure S1: Single-cell transcriptome profiling of PBMCs from AS patients (n=3) and healthy controls (n=3). The number of genes and UMIs detected per cell, and the percentage of mitochondrial (MT) genes were all within the normal range. Each dot represents a cell.

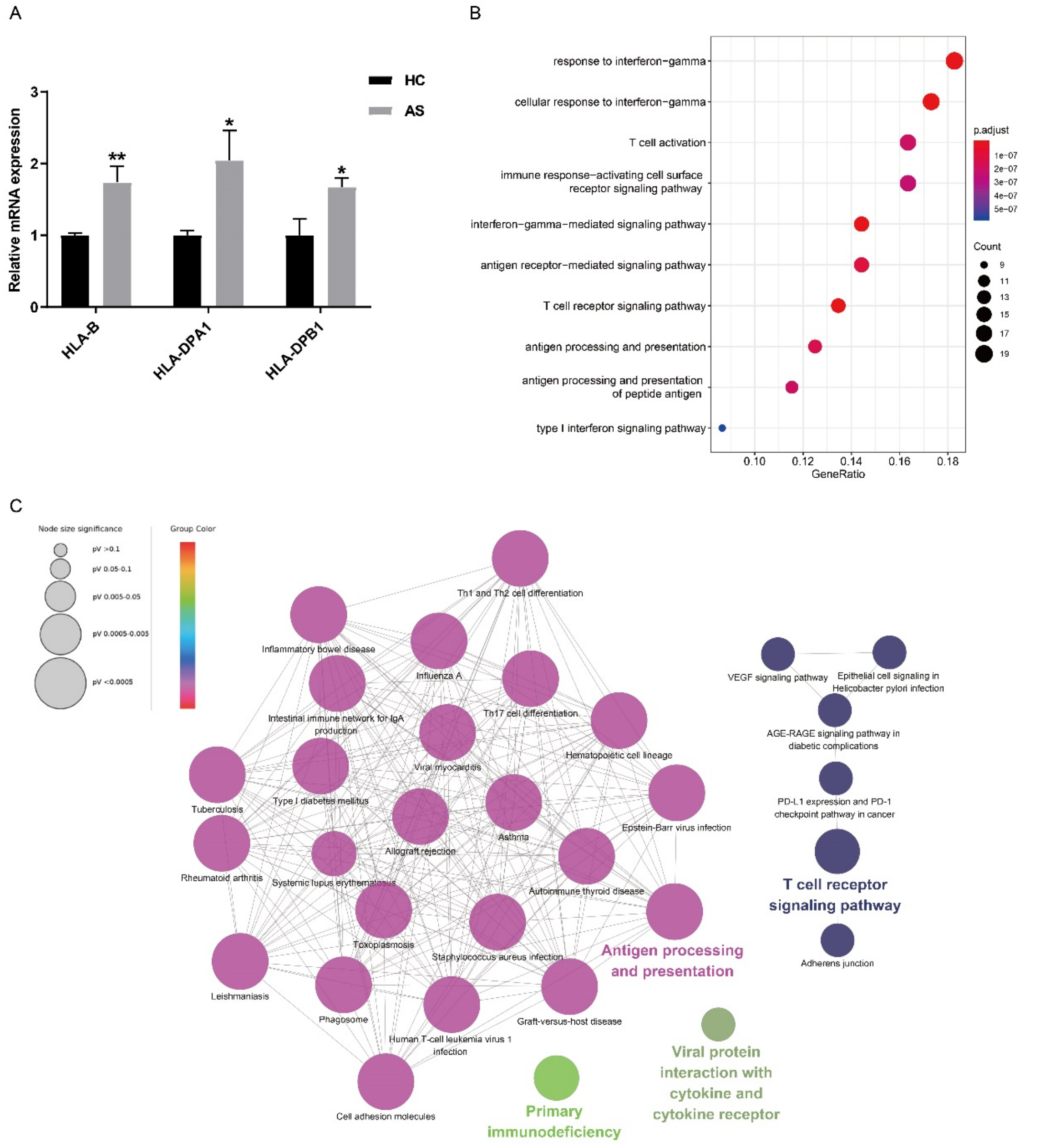

Figure S2: Validation and functional enrichment analysis of up-regulated DEGs in NK cells. (A) RT-qPCR analysis of gene expression fold changes in AS patients vs HCs. (B) Top 10 biological processes for up-regulated genes were shown in bubble plot according to gene ratio. (C) Use ClueGO plugin to analyze enriched KEGG pathways for up-regulated genes.
